# *N*,*N*′-Bis(2-amino­benz­yl)ethane-1,2-diaminium bis­(4-methyl­benzene­sulfonate)

**DOI:** 10.1107/S1600536811045879

**Published:** 2011-11-09

**Authors:** Luis Ángel Garza Rodríguez, Sylvain Bernès, Perla Elizondo Martínez, Blanca Nájera Martínez, Sara L. Rodríguez de Luna

**Affiliations:** a Laboratorio de Química Industrial, CELAES, Facultad de Ciencias Químicas, UANL, Pedro de Alba S/N, 66451 San Nicolás de los Garza, NL, Mexico; b DEP Facultad de Ciencias Químicas, UANL, Guerrero y Progreso S/N, Col. Treviño, 64570 Monterrey, NL, Mexico

## Abstract

The title salt, C_16_H_24_N_4_^2+^·2C_7_H_7_O_3_S^−^, crystallizes with the dication situated on an inversion center and the anion in a general position. The cation contains two ammonium and two free amine groups, and the observed conformation for the chain linking the benzene rings is different from that found in the free tetra­amine and in the fully protonated tetra­amine. All amine and ammonium H atoms of the cation form hydrogen bonds with eight symmetry-related anions, using the sulfonate O atoms as acceptors. This arrangement for the ions precludes any π–π contacts between benzene rings in the crystal.

## Related literature

For reviews on applications of macrocyclic systems, see: Vigato & Tamburini (2004 ▶); Radecka-Paryzek *et al.* (2005 ▶). For their acid-catalysed synthesis using *p*-toluene­sulfonic acid, see: Ionkin *et al.* (2008 ▶). For the structures of the free mol­ecule and the fully protonated cation corresponding to the title cation, see: Rodríguez de Barbarín *et al.* (2007 ▶) and Garza Rodríguez *et al.* (2009 ▶, 2011 ▶), respectively.
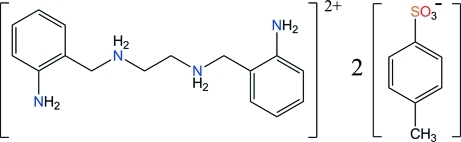


## Experimental

### 

#### Crystal data

C_16_H_24_N_4_^2+^·2C_7_H_7_O_3_S^−^
*M* *_r_* = 614.76
Triclinic, 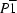

*a* = 5.753 (2) Å
*b* = 9.512 (3) Å
*c* = 14.493 (5) Å
α = 101.40 (2)°
β = 100.06 (3)°
γ = 97.80 (3)°
*V* = 753.6 (5) Å^3^
*Z* = 1
Mo *K*α radiation
μ = 0.23 mm^−1^
*T* = 298 K
0.60 × 0.16 × 0.16 mm

#### Data collection

Siemens P4 diffractometer
Absorption correction: ψ scan (*XSCANS*; Siemens, 1996 ▶) *T*_min_ = 0.512, *T*_max_ = 0.594
3505 measured reflections
2650 independent reflections
2234 reflections with *I* > 2σ(*I*)
*R*_int_ = 0.091
2 standard reflections every 98 reflections  intensity decay: 1%

#### Refinement

*R*[*F* ^2^ > 2σ(*F* ^2^)] = 0.056
*wR*(*F* ^2^) = 0.162
*S* = 1.29
2650 reflections
204 parameters
4 restraints
H atoms treated by a mixture of independent and constrained refinement
Δρ_max_ = 0.39 e Å^−3^
Δρ_min_ = −0.34 e Å^−3^

### 

Data collection: *XSCANS* (Siemens, 1996 ▶); cell refinement: *XSCANS*; data reduction: *XSCANS*; program(s) used to solve structure: *SHELXTL-Plus* (Sheldrick, 2008 ▶); program(s) used to refine structure: *SHELXTL-Plus*; molecular graphics: *SHELXTL-Plus* and *Mercury* (Macrae *et al.*, 2006 ▶); software used to prepare material for publication: *SHELXTL-Plus*.

## Supplementary Material

Crystal structure: contains datablock(s) I, global. DOI: 10.1107/S1600536811045879/fj2471sup1.cif

Structure factors: contains datablock(s) I. DOI: 10.1107/S1600536811045879/fj2471Isup2.hkl

Supplementary material file. DOI: 10.1107/S1600536811045879/fj2471Isup3.mol

Additional supplementary materials:  crystallographic information; 3D view; checkCIF report

## Figures and Tables

**Table 1:** Hydrogen-bond geometry (Å, °)

*D*—H⋯*A*	*D*—H	H⋯*A*	*D*⋯*A*	*D*—H⋯*A*
N1—H11⋯O2^i^	0.90 (1)	2.12 (1)	3.012 (3)	177 (3)
N1—H12⋯O3^ii^	0.91 (1)	2.27 (3)	3.028 (4)	141 (3)
N8—H81⋯O3^iii^	0.91 (1)	1.91 (2)	2.763 (3)	157 (3)
N8—H82⋯O1^iv^	0.91 (1)	1.86 (1)	2.739 (3)	160 (3)

Symmetry codes: (i) 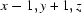; (ii) 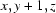; (iii) 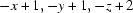; (iv) 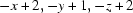.

**Fig. 1.**
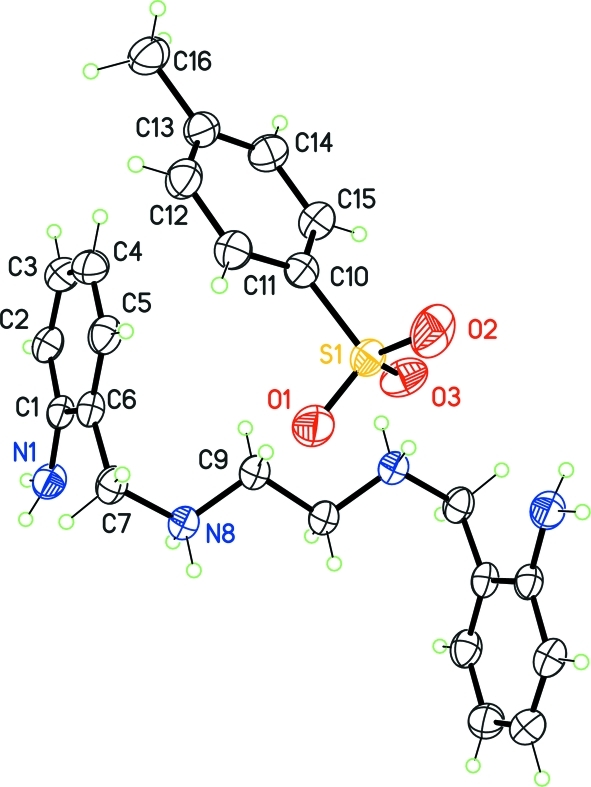
The structure of the title compound, with displacement ellipsoids at the 30% probability level. One cation and one anion are displayed. Non-labeled atoms are generated by symmetry code 1 - x, 1 - y, 2 - z.

**Fig. 2.**
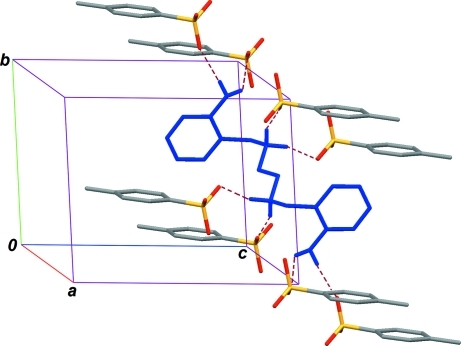
A part of the crystal structure of the title compound. The cation is displayed in blue, and is encapsulated by eight anions through hydrogen bonds (dashed lines).

## supplementary crystallographic information

### Comment

New routes for the preparation of macrocyclic crown ethers and related systems remains a topic of interest, because of their numerous potential applications, like recognizing and transporting specific metal ions, anions or neutral molecules (Vigato & Tamburini, 2004), modeling more intricate biological macrocyclic systems (hemoglobin, myoglobin, cytochromes, chlorophylls), corrins (vitamin B_12_) and antibiotics (valinomycin, nonactin), among others (Radecka-Paryzek *et al.*, 2005).

Macrocycles may be obtained through alkylation reactions, Mannich condensation, self condensation of nitriles, and Schiff condensation, the latter being an expeditive and very easy technique. Template and direct synthesis are however the two most popular methods for these synthesis. The template synthesis uses a metal ion to orient the reacting groups (primary amine and carbonyl) in a conformation suitable for ring closure. Direct synthesis may be carried out at *high dilution*, where the reactants are mixed slowly, keeping a concentration in the range 10^-2^–10^-3^*M*, or at *low dilution*, an acid-catalyzed route that uses very low concentrations of acids (10^-4^*M*) to emulate the natural acidity of the hydrolysis of metal ion. Acids commonly used are sulfuric, hydrochloric, hydrobromic, formic and *p*-toluenesulfonic acids (Ionkin *et al.*, 2008)

The title salt was obtained in a low yield as a by-product during the condensation reaction between 2,6-diacetylpyridine and *N*,*N*'-bis(*o*-aminobenzylidene)-1,2-diaminoethane, in the presence of *p*-toluenesulfonic acid (see *Experimental*). The asymmetric unit contains one-half cation, close to an inversion center, and one *p*-toluenesulfonate anion, placed in general position (Fig. 1). In the cation, amine groups of the 1,2-diaminoethane core are clearly protonated, while terminal NH_2_ groups remain unprotonated. The central chain linking benzene rings displays a *gauche*–*trans*–*trans*–*trans*–*gauche* conformation, defined by torsion angles C6—C7—N8—C9 [58.7 (3)°], C7—N8—C9—C9*^i^*, [171.5 (2)°], and N8—C9—C9*^i^*—N8*^i^* [180°, imposed by symmetry; symmetry code: (*i*) 1 - *x*, 1 - *y*, 2 - *z*]. This observed conformation is different from those displayed by the free tetraamine (*trans*–*gauche*–*trans*–*gauche*–*trans*; Rodríguez de Barbarín *et al.*, 2007) and by the tetracation [all-*trans*; Garza Rodríguez *et al.*, 2009, 2011]. The degree of protonation thus seems to influence the conformation stabilized in the solid-state for this system.

In the crystal structure, all N and O atoms are involved in hydrogen bonding, forming a three-dimensional supramolecular network. One cation is connected to eight symmetry-related anions, *via N*—*H*···*O*(sulfonate) hydrogen bonds (Fig. 2). Ammonium groups give hydrogen bonds of higher strength compared to contacts formed by amine NH_2_ groups. In the former case, O···H separations are short, *ca*. 1.9 Å, while in the latter they are rather long, *ca*. 2.2 Å.

### Experimental

A 100 ml flask was charged with 2,6-diacetylpyridine (163 mg, 1 mmol) in 35 ml of toluene. Under agitation at room temperature, *p*-toluenesulfonic acid monohydrate was added (38 mg, 0.20 mmol in 35 ml of toluene), and the mixture was further stirred for 30 min. A dissolution of *N*,*N'*-bis(*o*-aminobenzylidene)-1,2-diaminoethane (281 mg, 1.05 mmol, in 15 ml of toluene) was slowly added. The mixture was kept under these conditions for 2 days, affording a light yellow precipitate. The solid was filtered off, washed with cold toluene and diethylether, and air dried. The crude solid was dissolved in hot methanol and left overnight at 298 K for slow evaporation, affording the title salt (Yield: 32%; m.p. 458 K). Few crystals of limited quality were picked off from this material. Analysis found (calc. for C_30_H_38_N_4_O_6_S_2_): C 58.64 (58.61), H 6.67 (6.23), N 9.52 (9.12), S 10.20 (10.43%). IR spectrum features vibrations characteristic of the sulfonate group, at 1124 (ν_as_), 1011 (ν_s_) and 684 cm^-1^ (δ_s_).

### Refinement

Very few needle-shaped crystals were found to be suitable for X-ray study, and were almost all twinned samples. Data were collected on a small part of an irregular needle, which gave symmetric diffraction peaks, although intensity was rather low. Amine and ammonium H atoms were found in a difference map and refined freely, with N—H bond lengths restrained to 0.90 (1) Å (4 restraints). All C-bonded H atoms were placed in idealized positions, with C—H bond lengths fixed to 0.93 (aromatic), 0.97 (methylene), or 0.96 Å (methyl). Isotropic displacement parameters for H atoms were calculated from displacements of parent atoms: *U*_iso_(H) = 1.5*U*_eq_(carrier atom) for methyl, NH_2_ and NH_2_^+^ groups; *U*_iso_(H) = 1.2*U*_eq_(carrier C) for other H atoms.

### Crystal data

**Table:**

C_16_H_24_N_4_^2+^·2C_7_H_7_O_3_S^−^	*Z* = 1
*M**_r_* = 614.76	*F*(000) = 326
Triclinic, *P*1	*D*_x_ = 1.355 Mg m^−^^3^
Hall symbol: -P 1	Melting point: 458 K
*a* = 5.753 (2) Å	Mo *K*α radiation, λ = 0.71073 Å
*b* = 9.512 (3) Å	Cell parameters from 53 reflections
*c* = 14.493 (5) Å	θ = 4.8–12.3°
α = 101.40 (2)°	µ = 0.23 mm^−^^1^
β = 100.06 (3)°	*T* = 298 K
γ = 97.80 (3)°	Needle, colourless
*V* = 753.6 (5) Å^3^	0.60 × 0.16 × 0.16 mm

### Data collection

**Table:**

Siemens P4 diffractometer	2234 reflections with *I* > 2σ(*I*)
Radiation source: fine-focus sealed tube	*R*_int_ = 0.091
graphite	θ_max_ = 25.1°, θ_min_ = 2.2°
2θ/ω scans	*h* = −6→1
Absorption correction: ψ scan (*XSCANS*; Siemens, 1996)	*k* = −11→11
*T*_min_ = 0.512, *T*_max_ = 0.594	*l* = −17→17
3505 measured reflections	2 standard reflections every 98 reflections
2650 independent reflections	intensity decay: 1%

### Refinement

**Table:**

Refinement on *F*^2^	Secondary atom site location: difference Fourier map
Least-squares matrix: full	Hydrogen site location: inferred from neighbouring sites
*R*[*F*^2^ > 2σ(*F*^2^)] = 0.056	H atoms treated by a mixture of independent and constrained refinement
*wR*(*F*^2^) = 0.162	*w* = 1/[σ^2^(*F*_o_^2^) + (0.0785*P*)^2^ + 0.0881*P*] where *P* = (*F*_o_^2^ + 2*F*_c_^2^)/3
*S* = 1.29	(Δ/σ)_max_ = 0.001
2650 reflections	Δρ_max_ = 0.39 e Å^−^^3^
204 parameters	Δρ_min_ = −0.34 e Å^−^^3^
4 restraints	Extinction correction: *SHELXTL-Plus* (Sheldrick, 2008), Fc^*^=kFc[1+0.001xFc^2^λ^3^/sin(2θ)]^-1/4^
0 constraints	Extinction coefficient: 0.098 (15)
Primary atom site location: structure-invariant direct methods	

### Fractional atomic coordinates and isotropic or equivalent isotropic displacement parameters (Å^2^)

**Table:**

	*x*	*y*	*z*	*U*_iso_*/*U*_eq_	
N1	0.3160 (5)	0.8765 (2)	0.87317 (17)	0.0649 (6)	
H11	0.204 (5)	0.933 (3)	0.867 (3)	0.097*	
H12	0.457 (4)	0.939 (3)	0.897 (2)	0.097*	
C1	0.3118 (4)	0.7634 (3)	0.79652 (17)	0.0496 (5)	
C2	0.1190 (5)	0.7221 (3)	0.71864 (19)	0.0593 (6)	
H2A	−0.0057	0.7749	0.7166	0.071*	
C3	0.1086 (6)	0.6054 (3)	0.6448 (2)	0.0688 (7)	
H3A	−0.0235	0.5793	0.5939	0.083*	
C4	0.2906 (6)	0.5270 (3)	0.6453 (2)	0.0716 (8)	
H4A	0.2850	0.4487	0.5945	0.086*	
C5	0.4818 (5)	0.5656 (3)	0.72205 (19)	0.0618 (7)	
H5A	0.6057	0.5121	0.7227	0.074*	
C6	0.4955 (4)	0.6815 (2)	0.79821 (17)	0.0500 (6)	
C7	0.6967 (4)	0.7159 (3)	0.88352 (18)	0.0569 (6)	
H7A	0.7604	0.8195	0.8990	0.068*	
H7B	0.8240	0.6642	0.8679	0.068*	
N8	0.6196 (3)	0.6745 (2)	0.96988 (14)	0.0498 (5)	
H81	0.524 (5)	0.733 (3)	0.995 (2)	0.075*	
H82	0.749 (3)	0.700 (3)	1.0198 (15)	0.075*	
C9	0.5193 (5)	0.5189 (3)	0.95362 (18)	0.0566 (6)	
H9A	0.6282	0.4608	0.9270	0.068*	
H9B	0.3678	0.4963	0.9075	0.068*	
S1	0.81511 (10)	0.18163 (7)	0.85656 (4)	0.0543 (3)	
O1	0.9816 (4)	0.3162 (3)	0.89311 (16)	0.0927 (8)	
O2	0.9253 (5)	0.0558 (3)	0.85049 (17)	0.0967 (8)	
O3	0.6275 (4)	0.1732 (3)	0.90879 (14)	0.0786 (6)	
C10	0.6800 (4)	0.1845 (2)	0.73869 (17)	0.0488 (6)	
C11	0.8130 (5)	0.2400 (3)	0.67923 (18)	0.0572 (6)	
H11A	0.9744	0.2800	0.7023	0.069*	
C12	0.7067 (5)	0.2359 (3)	0.5859 (2)	0.0647 (7)	
H12A	0.7988	0.2732	0.5467	0.078*	
C13	0.4679 (5)	0.1784 (3)	0.54872 (19)	0.0587 (6)	
C14	0.3383 (5)	0.1247 (3)	0.6094 (2)	0.0640 (7)	
H14A	0.1766	0.0852	0.5863	0.077*	
C15	0.4404 (4)	0.1275 (3)	0.70353 (19)	0.0580 (6)	
H15A	0.3478	0.0911	0.7430	0.070*	
C16	0.3535 (6)	0.1760 (4)	0.4471 (2)	0.0793 (9)	
H16A	0.3956	0.2703	0.4341	0.119*	
H16B	0.1824	0.1518	0.4384	0.119*	
H16C	0.4092	0.1045	0.4037	0.119*	

### Atomic displacement parameters (Å^2^)

**Table:**

	*U*^11^	*U*^22^	*U*^33^	*U*^12^	*U*^13^	*U*^23^
N1	0.0860 (16)	0.0610 (14)	0.0559 (13)	0.0284 (12)	0.0228 (12)	0.0149 (11)
C1	0.0595 (13)	0.0503 (12)	0.0476 (13)	0.0149 (10)	0.0192 (10)	0.0202 (10)
C2	0.0606 (14)	0.0688 (16)	0.0572 (15)	0.0215 (12)	0.0131 (11)	0.0270 (13)
C3	0.0760 (17)	0.0695 (17)	0.0579 (16)	0.0132 (14)	0.0021 (13)	0.0175 (13)
C4	0.098 (2)	0.0614 (16)	0.0552 (16)	0.0206 (15)	0.0156 (15)	0.0083 (13)
C5	0.0751 (16)	0.0615 (15)	0.0611 (16)	0.0279 (13)	0.0250 (13)	0.0220 (12)
C6	0.0559 (13)	0.0521 (13)	0.0514 (13)	0.0148 (10)	0.0186 (10)	0.0236 (10)
C7	0.0516 (13)	0.0674 (15)	0.0587 (15)	0.0095 (11)	0.0165 (11)	0.0266 (12)
N8	0.0484 (10)	0.0550 (11)	0.0495 (12)	0.0109 (8)	0.0114 (8)	0.0177 (9)
C9	0.0661 (14)	0.0558 (14)	0.0510 (14)	0.0082 (11)	0.0143 (11)	0.0189 (11)
S1	0.0529 (4)	0.0600 (4)	0.0533 (4)	0.0159 (3)	0.0100 (3)	0.0175 (3)
O1	0.0863 (14)	0.0916 (16)	0.0797 (15)	−0.0221 (12)	−0.0219 (11)	0.0317 (12)
O2	0.1200 (19)	0.1028 (18)	0.0909 (16)	0.0728 (16)	0.0241 (14)	0.0392 (14)
O3	0.0748 (12)	0.1109 (17)	0.0519 (11)	0.0177 (12)	0.0235 (9)	0.0130 (10)
C10	0.0497 (12)	0.0487 (12)	0.0517 (13)	0.0177 (10)	0.0141 (10)	0.0112 (10)
C11	0.0548 (13)	0.0621 (15)	0.0584 (15)	0.0122 (11)	0.0178 (11)	0.0156 (11)
C12	0.0761 (17)	0.0680 (16)	0.0573 (16)	0.0164 (13)	0.0224 (13)	0.0215 (13)
C13	0.0716 (16)	0.0553 (14)	0.0526 (14)	0.0250 (12)	0.0104 (12)	0.0130 (11)
C14	0.0554 (14)	0.0754 (17)	0.0600 (16)	0.0168 (12)	0.0056 (11)	0.0148 (13)
C15	0.0511 (13)	0.0703 (16)	0.0576 (15)	0.0143 (11)	0.0152 (11)	0.0201 (12)
C16	0.105 (2)	0.0776 (19)	0.0568 (17)	0.0340 (18)	0.0050 (16)	0.0165 (14)

### Geometric parameters (Å, °)

**Table:**

N1—C1	1.380 (3)	C9—H9A	0.9700
N1—H11	0.896 (10)	C9—H9B	0.9700
N1—H12	0.907 (10)	S1—O2	1.423 (2)
C1—C2	1.388 (4)	S1—O3	1.425 (2)
C1—C6	1.395 (3)	S1—O1	1.435 (2)
C2—C3	1.367 (4)	S1—C10	1.756 (3)
C2—H2A	0.9300	C10—C15	1.377 (4)
C3—C4	1.365 (4)	C10—C11	1.381 (3)
C3—H3A	0.9300	C11—C12	1.374 (4)
C4—C5	1.373 (4)	C11—H11A	0.9300
C4—H4A	0.9300	C12—C13	1.378 (4)
C5—C6	1.380 (4)	C12—H12A	0.9300
C5—H5A	0.9300	C13—C14	1.376 (4)
C6—C7	1.487 (4)	C13—C16	1.500 (4)
C7—N8	1.503 (3)	C14—C15	1.381 (4)
C7—H7A	0.9700	C14—H14A	0.9300
C7—H7B	0.9700	C15—H15A	0.9300
N8—C9	1.471 (3)	C16—H16A	0.9600
N8—H81	0.905 (10)	C16—H16B	0.9600
N8—H82	0.912 (10)	C16—H16C	0.9600
C9—C9^i^	1.503 (5)		
			
C1—N1—H11	118 (2)	C9^i^—C9—H9A	109.6
C1—N1—H12	117 (2)	N8—C9—H9B	109.6
H11—N1—H12	105 (3)	C9^i^—C9—H9B	109.6
N1—C1—C2	121.3 (2)	H9A—C9—H9B	108.1
N1—C1—C6	120.5 (2)	O2—S1—O3	111.59 (15)
C2—C1—C6	118.1 (2)	O2—S1—O1	113.75 (17)
C3—C2—C1	121.5 (2)	O3—S1—O1	111.44 (16)
C3—C2—H2A	119.3	O2—S1—C10	107.00 (13)
C1—C2—H2A	119.3	O3—S1—C10	106.50 (12)
C4—C3—C2	120.5 (3)	O1—S1—C10	106.03 (12)
C4—C3—H3A	119.7	C15—C10—C11	119.1 (2)
C2—C3—H3A	119.7	C15—C10—S1	120.15 (19)
C3—C4—C5	118.8 (3)	C11—C10—S1	120.71 (19)
C3—C4—H4A	120.6	C12—C11—C10	120.0 (3)
C5—C4—H4A	120.6	C12—C11—H11A	120.0
C4—C5—C6	121.9 (2)	C10—C11—H11A	120.0
C4—C5—H5A	119.1	C11—C12—C13	122.1 (3)
C6—C5—H5A	119.1	C11—C12—H12A	119.0
C5—C6—C1	119.1 (2)	C13—C12—H12A	119.0
C5—C6—C7	121.1 (2)	C12—C13—C14	117.0 (3)
C1—C6—C7	119.7 (2)	C12—C13—C16	121.5 (3)
C6—C7—N8	112.20 (19)	C14—C13—C16	121.4 (3)
C6—C7—H7A	109.2	C13—C14—C15	122.1 (3)
N8—C7—H7A	109.2	C13—C14—H14A	118.9
C6—C7—H7B	109.2	C15—C14—H14A	118.9
N8—C7—H7B	109.2	C10—C15—C14	119.7 (2)
H7A—C7—H7B	107.9	C10—C15—H15A	120.2
C9—N8—C7	113.4 (2)	C14—C15—H15A	120.2
C9—N8—H81	113 (2)	C13—C16—H16A	109.5
C7—N8—H81	112.8 (19)	C13—C16—H16B	109.5
C9—N8—H82	110.1 (19)	H16A—C16—H16B	109.5
C7—N8—H82	108.0 (19)	C13—C16—H16C	109.5
H81—N8—H82	99 (3)	H16A—C16—H16C	109.5
N8—C9—C9^i^	110.5 (3)	H16B—C16—H16C	109.5
N8—C9—H9A	109.6		
			
N1—C1—C2—C3	−176.7 (2)	O3—S1—C10—C15	−23.7 (2)
C6—C1—C2—C3	−0.6 (4)	O1—S1—C10—C15	−142.5 (2)
C1—C2—C3—C4	−0.8 (4)	O2—S1—C10—C11	−82.4 (2)
C2—C3—C4—C5	1.2 (4)	O3—S1—C10—C11	158.2 (2)
C3—C4—C5—C6	−0.3 (4)	O1—S1—C10—C11	39.4 (2)
C4—C5—C6—C1	−1.1 (4)	C15—C10—C11—C12	−0.9 (4)
C4—C5—C6—C7	176.1 (2)	S1—C10—C11—C12	177.31 (19)
N1—C1—C6—C5	177.6 (2)	C10—C11—C12—C13	0.3 (4)
C2—C1—C6—C5	1.5 (3)	C11—C12—C13—C14	0.1 (4)
N1—C1—C6—C7	0.3 (3)	C11—C12—C13—C16	179.3 (2)
C2—C1—C6—C7	−175.8 (2)	C12—C13—C14—C15	0.0 (4)
C5—C6—C7—N8	−106.2 (3)	C16—C13—C14—C15	−179.3 (2)
C1—C6—C7—N8	71.0 (3)	C11—C10—C15—C14	1.0 (4)
C6—C7—N8—C9	58.7 (3)	S1—C10—C15—C14	−177.23 (19)
C7—N8—C9—C9^i^	171.5 (2)	C13—C14—C15—C10	−0.5 (4)
O2—S1—C10—C15	95.8 (2)		

Symmetry codes: (i) −*x*+1, −*y*+1, −*z*+2.

### Hydrogen-bond geometry (Å, °)

**Table:**

*D*—H···*A*	*D*—H	H···*A*	*D*···*A*	*D*—H···*A*
N1—H11···O2^ii^	0.90 (1)	2.12 (1)	3.012 (3)	177 (3)
N1—H12···O3^iii^	0.91 (1)	2.27 (3)	3.028 (4)	141 (3)
N8—H81···O3^i^	0.91 (1)	1.91 (2)	2.763 (3)	157 (3)
N8—H82···O1^iv^	0.91 (1)	1.86 (1)	2.739 (3)	160 (3)

Symmetry codes: (ii) *x*−1, *y*+1, *z*; (iii) *x*, *y*+1, *z*; (i) −*x*+1, −*y*+1, −*z*+2; (iv) −*x*+2, −*y*+1, −*z*+2.
